# Randomised controlled trial of welfare rights advice accessed via primary health care: pilot study [ISRCTN61522618]

**DOI:** 10.1186/1471-2458-6-162

**Published:** 2006-06-21

**Authors:** Joan Mackintosh, Martin White, Denise Howel, Tom Chadwick, Suzanne Moffatt, Mark Deverill, Adam Sandell

**Affiliations:** 1 Public Health Research Group, School of Population & Health Sciences, Faculty of Medical Sciences, University of Newcastle upon Tyne, NE2 4HH, UK

## Abstract

**Background:**

Little research has directly evaluated the impact of increasing financial or material resources on health. One way of assessing this lies with assisting people to obtain full welfare benefit entitlements. In 2000–1, 2.3 million pensioners were living in poverty in the UK and estimates suggest that around one million do not claim the financial support to which they are entitled. The effectiveness of welfare rights advice services delivered via primary health care to promote health and reduce health inequalities is unknown.

**Methods:**

The main objectives of this study were to assess the feasibility and acceptability of a randomised controlled trial of welfare rights advice in a community setting and identify appropriate health and social outcome measures in order to plan a definitive trial.

This was a single blind, community-based, pilot randomised controlled trial. 126 men and women aged 60 years and over, recruited from 4 general practices in Newcastle upon Tyne, UK, participated. The intervention comprised a structured welfare rights assessment followed by active assistance with welfare benefit claims over the following 24 months. The control group received the intervention after a six month delay. A range of socio-economic, health, behavioural and psycho-social outcomes were measured.

**Results:**

126 out of 400 people invited agreed to participate and 109 were followed up at 24 months. Both the intervention and research procedures were feasible and acceptable to participants and professionals involved. 68 (58%) of all participants received a welfare benefit award (31 financial, 16 non-financial and 21 both). Median time to receipt of benefits from initial assessment was 14 (range 1 to 78) weeks and median financial award was £55 (€81, $98) per household per week. There was little evidence of health-related differences between groups or over time, which could be due to limitations of the study design.

**Conclusion:**

Modification of the study design, including selection of study participants, timing of interventions and length of follow up are recommended for a definitive trial. More appropriate health and psycho-social outcome measures relevant to the elderly population should be sought, particularly focussing on those issues highlighted in the accompanying qualitative study.

## Background

Major inequalities in health result from differences in socio-economic position between individuals, families and population groups[1]. Whilst the link between resources and health is well established[1-4], there has been little conclusive research evaluating the impact of increasing resources on health[5]. In the UK large amounts of welfare benefits are unclaimed[6], particularly amongst vulnerable groups such as older people. It has been estimated that only around 40–60% of those eligible actually claim the health-related benefits to which they are entitled[7]. Appropriate targeting and active assistance with benefit claims can result in substantial increases in financial and non-financial resources (e.g. parking permits, household aids and adaptations) for eligible non-claimants[8-10]. In the UK welfare rights advice is offered through local government social services departments, Citizens Advice Bureaux (one of the UK's largest voluntary organisations, providing free advice and information on money, legal or other problems) or primary care[11], with clients accessing the services either through self-referral, referral from another agency or both. However, these services are not available to everyone.

Evaluating the health impact of complex social interventions poses ethical and methodological challenges. In particular, the ethical acceptability of withholding welfare advice or benefits from control group participants has been questioned[12]. Pilot studies play an important role in health research and are a necessary prerequisite for definitive randomised controlled trials[13,14]. Evaluations of the health effects of social interventions are essential to identify effective ways to reduce health inequalities[10,12].

We report the results of a pilot randomised controlled trial of welfare rights advice in a primary care setting, whilst Moffatt *et al*, in an accompanying paper[15], report the findings of an embedded qualitative study. We aimed to evaluate the methods, including a range of potential outcome measures, estimate effect sizes and sample size for a definitive trial.

## Methods

### Study design

We conducted a single blind RCT with individuals randomly allocated to intervention (receipt of immediate welfare rights assessment, advice and active assistance with claims) or control condition (receipt of the intervention after a six month delay).

### Recruitment

#### General practices

Four practices working from five premises in Newcastle upon Tyne participated. Three premises were in the top ten per cent of the most deprived wards in England and two were in the top one per cent, measured using the Index of Multiple Deprivation[16].

#### Participants

A random sample of 400 patients aged 60 years or over was generated using participating practices' computer systems, and invited to participate. Patients likely to have received full welfare assessments, such as the permanently hospitalised or those in residential or nursing care homes, were excluded. The sample size was pragmatic and aimed to enable us to estimate sample size for a future definitive trial.

### Intervention condition

Within 3 weeks of the baseline assessment, a welfare rights officer from Newcastle City Council Social Services undertook a structured assessment of current welfare status and benefits entitlement, including a full assessment of household income and expenditure. Participants were then offered active assistance with making benefit and other welfare claims where appropriate over the following months. Advice was offered either at home or at the GP surgery, but all participants preferred a domiciliary service.

### Control condition

Participants in the control group were given an appointment for a follow up interview with the researcher six months after their baseline assessment. Following this second interview, the control group were offered an appointment with the welfare rights officer, who offered a full welfare benefits assessment and active assistance with claims as appropriate, following the same procedure as the intervention group.

### Randomisation and blinding

Following the baseline assessment, participants were randomly allocated to the intervention or control group by the project secretary using a sequential allocation table independently generated from random number tables prior to recruitment. The researcher conducting the assessments was blind to the randomisation group.

### Baseline assessment and outcome measures

Written informed consent was obtained at baseline assessment. The following outcomes were collected in a structured, face-to-face interview at baseline, 6, 12 and 24 months:

#### Health

Short Form 36 (SF36)[17], Hospital Anxiety and Depression Scale (HADS)[18], activity limiting long term illness[19], symptoms inventory[20], the Pittsburgh Sleep Quality Index[21], and self-reported height and weight (used to calculate Body Mass Index (weight in kg/height in m^2^)).

#### Health related behaviours

The Dietary Inventory for Nutrition Education (DINE),[22] the Physical Activity Scale for the Elderly (PASE)[23], current smoking status and weekly alcohol consumption[24].

#### Psycho-social factors

Social Support Questionnaire[25], the Self-Esteem Inventory[26], the Personal Mastery Scale[27] and the Life Events Inventory[20].

#### Socio-economic status

Affordability (financial vulnerability)[28] and Standard of Living Index[29].

In addition data were collected at baseline on age, sex, ethnicity and educational attainment. The salary, travel and administrative costs of providing welfare rights advice were estimated.

Participants were contacted by telephone for repeat welfare assessments at 6, 12 and 24 (intervention) or 6 and 18 months (control) from the initial welfare assessment. When an application for benefits was made, the Welfare Rights Officer asked participants to notify her when a decision letter arrived, so that an accurate record could be kept of benefits received. Participants who had not notified her were followed up with telephone calls.

Intervention cost per case was estimated by recording the number of minutes spent on each participant's case by the Welfare Rights Officer, including advice sessions, telephone calls and letter writing, and recording the number of miles travelled. Travel was costed at £0.404 (€0.595, $0.716) per mile, and staff time at £0.934 (€1.374, $1.656) per minute. Household income and major expenditure (housing costs, council tax, loan repayments and energy costs) were used to estimate household disposable income.

### Statistical methods

Initial analyses were conducted on an 'intention-to-treat' basis, without regard to whether participants had received benefits as a result of the intervention. Explanatory analyses then compared outcomes in the intervention at different time points.

The differences between mean changes from baseline in each outcome measure for the intervention and control groups are reported along with 95% confidence intervals for those differences. Bootstrap methods were used when the distributions were markedly non-normal. ANOVA was used to compare outcomes at different time points.

### Ethical approval

The protocol for the study was approved by Newcastle upon Tyne joint universities and NHS research ethics committee.

## Results

### Participant flow and follow-ups

**Figure 1:**
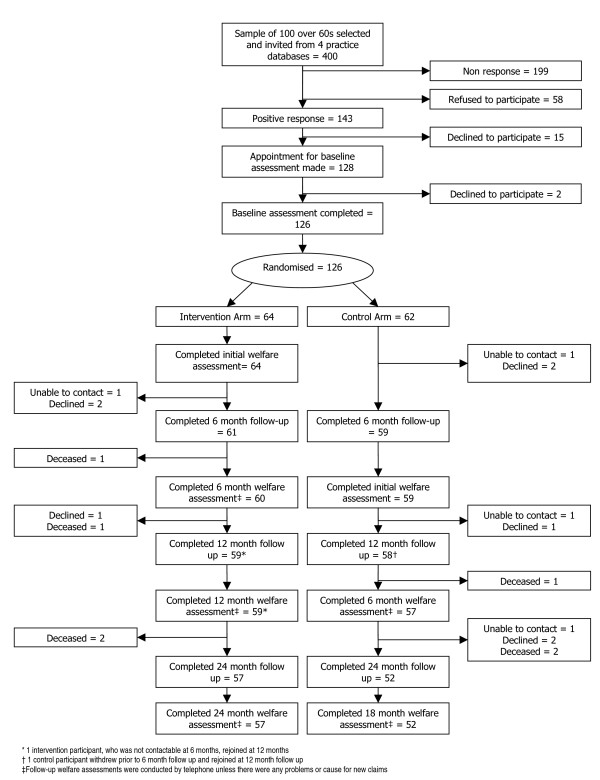
Participant flow and follow-up.

Out of the 400 people invited to participate, 199 (49.8%) did not respond, and 58 (14.5%) declined to participate. Of the latter, 30% gave no reason, 32% said they were not interested or felt they would be ineligible for welfare benefits and 39% cited being too old, sick or frail. Between August and December 2002, 126 people were recruited and randomised (figure 1). Characteristics of the intervention and control groups were similar at baseline (table 1). SF-36 scores showed poorer physical health than expected for their ages, although mental health scores were within normal range[17]. There was little drop out in subsequent assessments (figure 1).

### Material and financial outcomes

Table 2 shows the distribution of initial disposable income and any awards made to participants, along with the period they had to wait for financial benefits. Sixty eight (58%) of all participants received a welfare benefit award (31 financial, 16 non-financial and 21 both). Non-financial awards included the disabled parking permit ("blue badge"), aids and adaptations around the home, "Staywarm"- a national energy scheme, and the Community Care Alarm scheme. Benefit eligibility was greater in general practices with higher levels of deprivation, measured using the Index of Multiple Deprivation[16] (table 3).

**Figure 2:**
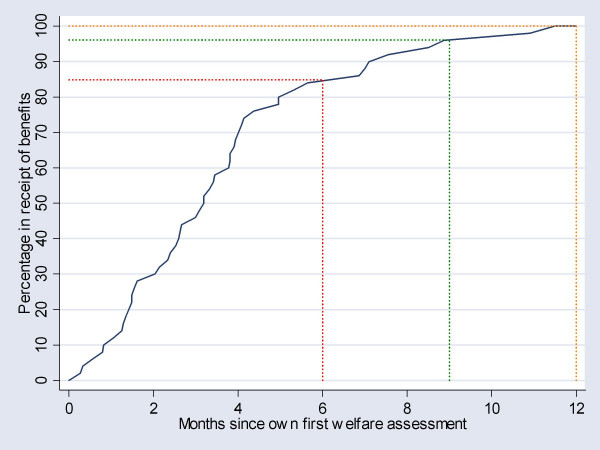
Time from own first assessment to receiving financial benefits in intervention and control groups combined.

Median time to receipt of benefits from initial assessment overall was 14 (range 1 to 78) weeks and median financial award was £55 (€81, $98) per household per week. At 6 months after the initial welfare assessment, eligible participants had been in receipt of benefits for a median of 9 weeks but seven (14%) had not yet received their benefits (table 2). However, all participants had received their benefits by 12 months (figure 2).

The mean intervention cost per case was £120.18 (€172.34, $209.94). This comprised salary of £115.24 (€165.20, $201.21) and travel of £4.94 (€7.08, $8.62). The mean cost was £161.23 (€231.14, $281.46) for those who received benefits and £63.62 (€91.21, $111.07) for those who did not.

### Health, behavioural and psychosocial outcomes

There was considerable variability in the change of most outcomes at 6 months (table 4) but the mean change was close to zero for most scales. The distributions of the changes over 6 months were very similar for both groups. The only significant difference in mean change was for the financial vulnerability score; those in the intervention group felt less vulnerable than those in the control group at six months (difference = -1.6 (95% CI: -2.6 to -0.7)). However, the change on this scale was zero for most participants.

Since welfare benefits did not always start until late in the initial follow-up period, 6 months may have been too early to detect any health-related improvements. However, the mean outcome measures varied very little across 6, 12 and 24 month time points in the intervention group, apart from sleep quality and social interaction, which both improved between 6 and 12 months and declined between 12 and 24 months (table 5).

## Discussion

### Main findings

This is the first pilot RCT to measure a broad range of health and social outcomes in relation to a welfare rights advice intervention accessed via a health care setting. Both the intervention and research procedures proved feasible and acceptable to participants and professionals involved.

Around 60% of participants were eligible for some benefits and 40% for financial benefits, confirming that offering welfare advice to older people via primary care is an effective way of identifying those entitled to benefits who would otherwise be unlikely to claim[8,9,30]. However, there was little evidence of differences in health outcomes between those who did or did not receive the advice at 6 months, or within the intervention group over time. These could reflect a genuine lack of effect, or limitations in the study design; this was a pilot study and not powered to detect small differences. Previous studies examining the impact of welfare rights advice on health have been observational and therefore provide only weak evidence[10,31]. Our qualitative study[15] suggests that those in receipt of extra benefits were more able to participate in society and experienced greater 'peace of mind', but this was not evident using scales such as Social Interaction[25] or HAD-Anxiety[18] in this pilot trial.

### Strengths and weaknesses

The study was conducted in the UK and the findings should therefore be interpreted with caution in relation to the health and welfare systems of other countries. Nevertheless, most of the implications are methodological in nature and thus widely applicable.

The methodological and ethical challenges in evaluating an intervention of this type have been discussed before[12], but the low drop-out rate suggests that participants found our study procedures acceptable, a finding corroborated by the accompanying qualitative study[15]. This was a single blind RCT and the letter arranging the follow up appointment specifically asked the participant not to tell the researcher whether or not they had seen the welfare adviser. Only one participant told the researcher conducting follow-up interviews (JM) that she had received benefits, but this was an isolated incident and we do not believe that it affected the results. Furthermore, the main analyses were conducted by independent statisticians (DH, TC) who were blind to this information.

Although the health and social assessment interview covered a wide range of outcome measures, taking between 30 and 90 minutes to complete, participants did not report feeling this was too onerous a task for them.

Whilst the intervention in this study was the provision of a welfare rights assessment, in 40% of the sample this did not result in any welfare benefits because of ineligibility. This will have diluted any resultant health improvements in the intention to treat analysis. Using practice deprivation scores to target the poorest areas would increase the proportion of participants eligible for welfare benefits in a future trial. Further exploration of individual predictors of eligibility using GP data (e.g. morbidity indicators) in this study was hindered by the small sample size. It may be possible in future work to improve recruitment materials to persuade others likely to gain from a welfare rights assessment to participate. Thirty nine percent of those giving a reason for non-participation said that it was because they were too old, ill or frail. Whilst this is not uncommon in research with the elderly, these are likely to be people who could benefit from a welfare rights assessment. In contrast, the embedded qualitative study[15] indicated that some participants agreed to take part in this research study out of a sense of altruism, rather than because they thought they might qualify for welfare benefits. All participants reported their ethnic group to be white British. Whilst Newcastle overall has a small ethnic population, one of the study practices has a sizeable proportion of patients from the South Asian community. It is not known how many people from this community were invited to participate and either did not respond or declined. However, the invitation to participate was only available in English and this is likely to have affected recruitment of people for whom English was not their first language.

The potential health-related impact of additional benefits is hard to quantify. Some concerns were raised about the standard scales used to assess health related outcomes. Interviewer feedback suggested that some of the questions on the scales we used may have been inappropriate for this population; most were not designed specifically for use with older people and, additionally, some may not be sufficiently responsive to small changes in health which would be of interest. A related issue arose in interviews with the 14 (out of the sample of 25) people in the qualitative study[15] who had received financial benefits. A positive and wide-ranging impact on a number of aspects of quality of life such as 'maintaining independence' or 'peace of mind' was reported. These are all factors relevant to an older population but not specifically measured by the scales used in the RCT.

The optimum time at which to measure any health benefits was unclear at the trial outset. The six month delay prior to the control group's benefit assessment was thought to be a reasonable compromise between impeding the receipt of benefit entitlements and allowing time for any health improvements to appear. This was justified ethically on the grounds that the intervention is presently rationed and recipients would not have been identified within the welfare rights officer's normal caseload. However, 14% of those eligible for financial benefits did not receive them until after the six month follow up assessment, and the average time between receipt of financial benefits and the next assessment was only two months. Health improvements resulting from increased benefits may not be detectable after such a short time. The time lapse needed for additional resources to have any effect on health is unclear, but there is a methodological argument for delaying the welfare rights advice longer in the control group in a future trial. However, this remains ethically contentious, as it is hard to justify withholding an intervention that is known to be beneficial in financial terms[12].

## Conclusion

Our trial design was feasible and acceptable[15]. The study found a large proportion of participants in the sample was eligible for welfare benefits but not claiming them. However, there was little evidence of differences in health outcome measures between groups or over time. If there was a real effect, possible reasons for a lack of evidence include: the small sample size; inadequate lengths of time for additional welfare benefits to have health and psycho-social effects; many participants did not qualify for any benefits; and the outcome measures used may not have been the most appropriate. These factors combined would have reduced the observed strength of any possible effects.

Modification of the study design to reduce the dilution effects described above, including selection of study participants, timing of interventions and length of follow up will be necessary for a definitive trial. There is also a need to look at some alternative or additional measures of health outcomes relevant to an older population, particularly those highlighted by the accompanying qualitative study[15], such as maintenance of independent living : however many of the outcome measures used in this pilot RCT would remain relevant. This study provides an example of how a pilot RCT with an embedded qualitative study identified unforeseen problems that will inform the design of a definitive evaluation.

## Competing interests

The author(s) declare that they have no competing interests.

## Authors' contributions

MW and SM had the original idea for the study, conducted preliminary work and, with the help of DH, AS, Nick Whitton and Rosemary Bell, developed the proposal for this study and gained funding. Rosemary Bell and Jenny Dover delivered the intervention and AS, JM and SM briefed general practices. JM and RB collected all data. Laura Stokoe and JM prepared the data for analysis. JM, DH, TC and MW, with help from MD, AS and Jean Adams, analysed the RCT data. JM, DH and MW drafted the paper and received critical comments from AS and SM. All authors approved the final version. All authors are guarantors and accept full responsibility for the conduct of the study and contents of this paper.

## Pre-publication history

The pre-publication history for this paper can be accessed here:



## Figures and Tables

**Table 1:** Baseline characteristics of intervention and control groups

	**Intervention (n = 64)**	**Control (n = 62)**
	
	**Mean (SD)**	**Mean (SD)**
Age	70.8 (7.1)	70.4 (8.3)
**Health Status**		
SF36 – Physical (Range* 0–100, higher score = better health)	34.2 (10.9)	35.1 (11.4)
SF36 – Mental (Range 0–100, higher score = better health)	51.5 (8.6)	51.4 (8.6)
HAD – Anxiety (Range 0–21, lower score = less anxiety)	6.1 (3.9)	5.6 (4.4)
HAD – Depression (Range 0–21, lower score = less depression)	4.6 (3.5)	4.4 (3.2)
Sleep quality (PSQI) (Range 0–21, lower score = better sleep quality)	7.5 (4.2)	7.6 (3.7)
Symptom inventory (Range 0–21, lower score = fewer symptoms)	6.7 (3.6)	5.5 (3.1)
**Health related behaviours**		
Fruit and vegetable intake (Range 0–42, high score = higher intake)	18.6 (7.2)	19.4 (6.4)
Protein intake (Range 0–36, high score = higher intake)	8.8 (2.9)	9.2 (3.2)
Starch intake (Range 0–18, high score = higher intake)	14.0 (3.9)	14.5 (3.6)
Fat intake (Range 0–24, high score = higher intake)	10.4 (5.2)	11.2 (5.7)
Alcohol (units, high score = higher intake)	13.3 (18.6)	10.6 (12.9)
BMI (weight (kg)/height (m)^2^)	29.0 (4.8)	28.9 (5.1)
Physical activity (PASE) (Range 0–3503, high score =more physical activity)	82.7 (41.1)	80.8 (47.6)
**Psycho-Social Characteristics**		
Social interaction (Range 0–30, high score = high level of social interaction)	11.9 (4.3)	11.1 (4.5)
Strength of confiding relationships (Range 2–10, high score = strong relationships)	8.1 (2.3)	8.2 (2.1)
Self Esteem Inventory** (Range 10–40, high score = high self esteem)	30.8 (3.9)	30.8 (3.6)
Personal Mastery Scale** (Range 7–28, high score = high personal mastery)	19.8 (2.8)	19.9 (3.1)
Life Events Inventory (Range 0–32, high score = more stressful life events)	1.4 (1.4)	0.7 (1.0)
**Economic Status**		
Financial vulnerability (Range 6–28, lower score = less financially vulnerable)	8.7 (4.7)	7.4 (2.7)
Standard of Living Index (Range 0–23, high score = better standard of living)	16.3 (2.5)	16.3 (3.0)
	**n (%)**	**n (%)**
Sex (Male)	38 (59.4)	33 (53.2)
Education post 16 yrs	4 (6.3)	7 (11.3)
Ethnic group – White	64 (100.0)	62 (100.0)
Use of car	34 (53.1)	34 (54.8)
Long term limiting illness	41 (64.1)	40 (64.5)
Current smoker	12 (18.8)	12 (19.4)

* The range of the measurement scale

** 1 person in the intervention group did not complete self esteem/mastery assessment

**Table 2:** Distribution of household income, welfare benefit variables and type of award by group allocation

	**Intervention (n = 59)**	**Control (n = 58)**	**Total (n = 117)**
**Distribution of household income and welfare benefit variables**	**Median (Min, Max)**	**Median (Min, Max)**	**Median (Min, Max)**

Initial weekly disposable household Income in £	190 (40, 782)	195 (-12*, 711)	190 (-12*, 782)
Amounts of additional benefits received per week in £	60 (10, 118)	38 (4, 137)	55 (4, 137)
Total arrears received as a lump sum per household in £†	563 (50, 3627)	338 (70, 3935)	483 (50, 3935)
			
Time between welfare assessment and start of financial benefit in weeks	16 (1, 78)	10 (1, 39)	14 (1, 78)
Time between start of financial benefit and first follow-up health questionnaire after welfare assessment in weeks **‡	11 (-26, 24)	8 (-15, 23)	9(-26, 24)
			
**Type of Award**	**No. (%)**	**No. (%)**	**No. (%)**
Received only financial award(s)	12 (20.3)	19 (32.8)	31 (26.5)
Received only a non-financial award(s)	10 (16.9)	6 (10.3)	16 (13.7)
Received both a financial and non-financial award	12 (20.3)	9 (15.5)	21 (17.9)
Received some type of award	34 (57.6)	34 (58.6)	68 (58.1)
Received no award(s)	25 (42.4)	24 (41.4)	49 (41.9)

* One individual did not know their partner's pension, so the minimum household disposable income is an underestimate

** For those participants who received extra financial benefits (n = 24 in intervention group, n = 26 in control group).

† For those participants who received extra financial benefits (n = 19 in intervention group, n = 19 in control group).

‡ The values are negative if benefits were awarded after 6 month follow-up (if in intervention group) or 12 month follow-up (if in control group)

**Table 3:** Eligibility for financial benefits and median amount awarded by general practice

**Practice**	IMD* Score	IMD* Ranking	**% eligible for financial benefits**	**Median amount of benefit awarded/week (£)**	**IQR**
A/A1**	64.4/45.2	103/803	52	57	38–79
**B**	75.6	30	38	48	21–80
**C**	74.6	36	47	60	23–90
**D**	18.9	3774	24	16	11–24

*Index of Multiple Deprivation (DETR).[16] Ranking scores: 1 = most deprived, 8414 = least deprived.

** Practice A was a split site practice so the scores for both sites are given.

**Table 4:** Changes in continuous outcome measures^# ^from baseline to 6 months for intervention and control groups

	**Intervention (n = 61)**	**Control (n = 59)**	**Difference between means (95% CI)**
		
	**Mean Change (6m-baseline) (SD)**	**Mean Change (6m-baseline) (SD)**	
**Health Status**			
SF36 – Physical	1.6 (6.9)	0.2 (6.3)	1.4 (-1.0 to 3.8)
SF36 – Mental	0.3 (6.8)	-1.1 (7.0)	1.4 (-1.1 to 3.9)
HAD – Anxiety *	0.1 (2.7)	-0.6 (2.8)	0.7 (-0.3 to 1.7)
HAD – Depression *	0.4 (2.4)	0.3 (2.4)	0.1 (-0.8 to 1.0)
Sleep quality (PSQI)	2.3 (2.8)	2.0 (2.4)	0.3 (-0.6 to 1.2)
Symptom inventory	0 (2.6)	0.5 (2.4)	-0.5 (-1.4 to 0.4)
**Health related behaviours**			
Fruit and vegetable intake	-5.8 (6.4)	-5.8 (6.3)	0.0 (-2.3 to 2.3)
Protein intake	-3.1 (3.8)	-3.7 (3.7)	0.6 (-0.7 to 2.0)
Starch intake	-1.7 (2.9)	-2.2 (3.7)	0.6 (-0.6 to 1.8)
Fat intake	-2.3 (5.1)	-2.8 (4.7)	0.5 (-1.3 to 2.3)
Alcohol (units)	-1.9 (10.8)	-1.1 (8.7)	-0.8 (-4.3 to 2.8)
BMI	0.2 (1.2)	0.1 (1.6)	0.1 (-0.4 to 0.6)
Physical activity (PASE)	-0.6 (41.1)	-0.4 (47.4)	-0.1 (-16 to 16)
**Psycho-social outcomes**			
Social interaction	-0.2 (3.1)	-0.3 (3.0)	0.1 (-1.0 to 1.2)
Strength of confiding relationships	0.2 (1.2)	0.1 (1.7)	0.1 (-0.5 to 0.6)
Self Esteem Inventory *	-0.4 (2.7)	0.4 (3.2)	-0.8 (-1.9 to 0.3)
Personal Mastery Scale*	0.1 (1.9)	0.5 (2.5)	-0.4 (-1.2 to 0.4)
**Economic Status**			
Financial vulnerability **	-1.5 (3.4)	0.1 (1.8)	-1.6 (-2.6 to -0.7)
Standard of Living Index	0.3 (1.0)	0.4 (0.9)	-0.1 (-0.5 to 0.2)

* 1 person in the intervention group did not complete this scale

** 2 people in the intervention and 1 in the control group did not complete this scale

# A description of the scales used is given in Table 1.

**Table 5:** Comparison of mean outcome measure scores^# ^at 6, 12 & 24 months – Intervention group (n = 57)

	**6 months**	**12 months**	**24 months**	
	**Mean**	**Mean**	**Mean**	P-value*

SF36 – Physical	28.6	28.7	27.6	0.68
SF36 – Mental	51.7	53.0	52.3	0.23
HAD – Anxiety	6.3	6.2	6.6	0.38
HAD – Depression	4.7	4.5	4.9	0.15
BMI	29.2	29.1	29.1	0.74
Sleep Quality (PSQI)	10.0	8.7	11.2	<0.001
Social Interaction	11.9	12.8	12.2	0.02
Strength of Confiding Relationships	8.2	8.4	8.1	0.38
Self Esteem Inventory	30.6	30.8	31.0	0.33
Personal Mastery Scale	19.8	19.6	19.6	0.60
Financial Vulnerability	7.4	7.7	7.4	0.55

* From repeated measures ANOVA

# A description of the scales used is given in Table 1.
